# Reconsolidation of a well-learned instrumental memory

**DOI:** 10.1101/lm.035543.114

**Published:** 2014-09

**Authors:** Marc T.J. Exton-McGuinness, Rosemary C. Patton, Lawrence B. Sacco, Jonathan L.C. Lee

**Affiliations:** 1 School of Psychology, University of Birmingham, Edgbaston, Birmingham, B15 2TT, United Kingdom

## Abstract

Once consolidated, memories are dynamic entities that go through phases of instability in order to be updated with new information, via a process of reconsolidation. The phenomenon of reconsolidation has been demonstrated in a wide variety of experimental paradigms. However, the memories underpinning instrumental behaviors are currently not believed to reconsolidate. We show that well-learned lever pressing in rats does undergo reconsolidation, which can be disrupted by systemic administration of the noncompetitive *N*-methyl-D-aspartate receptor (NMDAR) antagonist (+)-5-methyl-10,11-dihydro-SH-dibenzo[a,d]cyclohepten-5,10-imine maleate (MK-801) when administered prior to a switch to a variable, but not fixed, ratio schedule. Disruption of reconsolidation resulted in a reduction in long-term lever pressing performance and diminished the sensitivity of behavior to contingency change. Further investigation demonstrated that expression of the reconsolidation impairment was not affected by outcome value, implying a deficit in a stimulus–response (S–R) process. The ability to disrupt the performance of well-learned instrumental behaviors is potentially of great importance in the development of reconsolidation-based clinical treatments for conditions that involve compulsive seeking behaviors.

Following initial acquisition, new memories exist in an unstable state; they are short-lasting and vulnerable to disruption by amnestic interventions. In order to persist in the long term, new memories must undergo a protein synthesis dependent process of consolidation (McGaugh 2000), which stabilizes the memory trace. Consolidated memories are not permanently fixed, however, and can be destabilized, becoming malleable and again vulnerable to amnestic treatment (Misanin et al. 1968). In order to be maintained, a process of reconsolidation is needed for memories to be returned to their stable form (Przybyslawski and Sara 1997; Nader 2003). While, experimentally, reconsolidation is typically disrupted in order to weaken memories, it is currently believed that reconsolidation normally functions to maintain memory relevance by updating their content (Lee 2009, 2010).

Reconsolidation has been demonstrated in a variety of experimental paradigms (for recent review, see Reichelt and Lee 2013a). However, well-learned instrumental memories are not believed to undergo reconsolidation (Hernandez and Kelley 2004; Mierzejewski et al. 2009). The precise parameters of the reactivation session appear to be a key factor in determining whether reconsolidation occurs, and it may be that the reactivation parameters used in past studies of instrumental reconsolidation have been inappropriate, or insufficient, to induce reconsolidation. Indeed, it has been proposed that differences in reactivation parameters may be the cause of other conflicting findings within the field (Piñeyro et al. 2014).

Many reactivation parameters have been shown to act as boundary conditions which constrain the reconsolidation process, such as the age (Suzuki et al. 2004) and strength of a memory (Suzuki et al. 2004; Reichelt and Lee 2012). In Pavlovian paradigms, a minimum amount of stimulus exposure appears to be required for reconsolidation to occur (Reichelt and Lee 2012), while excess exposure leads to extinction (Lee et al. 2006; Flavell and Lee 2013). Furthermore, intermediate levels of stimulus exposure may trigger neither reconsolidation nor extinction (Flavell and Lee 2013; Merlo et al. 2014). Therefore alteration of reward contingency, rather than absolute nonreinforcement, may be a more robust method of destabilizing memories, particularly when behavior is well-learned (Díaz-Mataix et al. 2013; Sevenster et al. 2013).

An additional confound in the investigation of instrumental memory is that behavior may be mediated via either a goal-directed, response–outcome (R–O) association (Balleine and Dickinson 1998; de Wit and Dickinson 2009), or an automated S–R habit (Dickinson 1985; Balleine and O'Doherty 2010). Furthermore Pavlovian and incentive processes can exert motivational effects on the vigor of performance (Dickinson and Balleine 1994; Rescorla 1994; Holland 2004). With limited training, behavior tends to fall under goal-directed control, transitioning to a habit with extended training (Dickinson 1985; Dickinson et al. 1995); however, this appears to depend upon the training schedule (Dickinson et al. 1983; Wiltgen et al. 2012). It is generally accepted that well-learned, habitual behaviors are less sensitive to the consequences of action (Balleine and Dickinson 1998), and to changes in the value of the behavioral outcome (Adams 1982; Dickinson 1985). On the other hand, sensitivity to outcome value and the consequences of a response is considered the canonical demonstration of goal-directed behavior (Balleine and Dickinson 1998; Balleine and O'Doherty 2010). It remains unclear whether destabilization of a well-learned instrumental memory would result in reconsolidation of one or both of these processes.

In the present study, we tested several reactivation parameters for their ability to destabilize an instrumental lever pressing memory. We chose to use NMDAR antagonism, rather than protein synthesis inhibition, to disrupt reconsolidation, as protein synthesis inhibitors have secondary effects upon incentive processes due to the general malaise they cause (Jonkman and Everitt 2009, 2011). This can impair motivation to acquire the reinforcer, leading to reductions in lever pressing performance that are not mediated by weakening of memory (Hernandez and Kelley 2004). NMDAR antagonism is frequently used to disrupt memory reconsolidation (Lee et al. 2006; Milton et al. 2008a), and in the case of appetitive paradigms may be a universal requirement of reconsolidation (Reichelt and Lee 2013a). Following the successful disruption of reconsolidation we probed the sensitivity of remaining behavior to reward devaluation and changes in contingency, in order to determine the nature of the impairment.

## Results

### Brief nonreinforced reactivation

We first confirmed that our training protocol produced habitual behavior, as assessed by sensitivity to outcome devaluation (Supplemental Fig. S1). We then tested whether a brief, nonreinforced reactivation session could successfully destabilize lever pressing memory. Short extinction sessions are commonly used in reconsolidation studies to destabilize memories (Lee et al. 2006; Milton et al. 2012; Flavell and Lee 2013).

**Figure 1.**
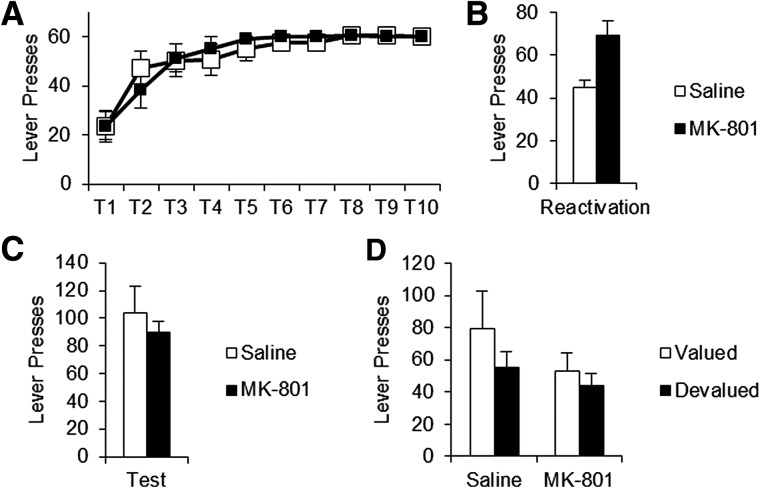
A brief nonreinforced reactivation session was ineffective in destabilizing instrumental memory. (*A*) During training both saline (white squares) and MK-801 (black squares) drug groups displayed equivalent levels of lever pressing. Training sessions were capped at a maximum of 60 rewards. (*B*) During the brief nonreinforced reactivation, MK-801-treated rats (*n* = 12) displayed significantly elevated lever pressing compared to saline controls (*n* = 12). (*C*) When tested 24 h after reactivation, there was no significant difference in lever pressing between treatment groups. (*D*) The outcome was then valued (white bars) or devalued (black bars) and behavior retested (final *n* = 6 per condition). Lever pressing did not display any significant sensitivity to reward devaluation, regardless of previous drug treatment. Data expressed as mean ± SEM.

Rats significantly increased their lever pressing over the 10 d of Training (*F*_(2.82,62.04)_ = 21.61, *P* < 0.001) (Fig. 1A) with no significant differences between treatment groups (Treatment, *F*_(1,22)_ = 0.02, *P* = 0.898; Training × Treatment, *F*_(2.82,62.04)_ = 0.59, *P* = 0.614).

Following training, rats were injected with MK-801, or saline vehicle, 30 min prior to experiencing the brief nonreinforced reactivation. During reactivation, MK-801-injected rats showed significantly elevated lever pressing performance compared to saline controls (*F*_(1,22)_ = 10.52, *P* = 0.004) (Fig. 1B). However, there was no significant long-term effect of drug treatment 24 h later during an extinction test (*F*_(1,22)_ = 0.464, *P* = 0.503) (Fig. 1B). Nosepoking behavior did not significantly differ between treatment groups at any session (Supplemental Fig. S2).

### Reward devaluation

As instrumental behavior can be mediated via one of two processes; it has been suggested that a reconsolidation impairment in S–R memory could be masked by compensation from an intact R–O association (Milton and Everitt 2012). In order to test this, we devalued the sucrose pellets by pairing them with LiCl injection. Groups were similarly performing on the first test prior to devaluation (Supplemental Fig. S3A). Rats were then retested in extinction (Fig. 1D). Lever pressing did not show any significant sensitivity to outcome value (*F*_(1,20)_ = 1.31, *P* = 0.266), nor was there a long-term effect of previous MK-801 injection (*F*_(1,20)_ = 1.65, *P* = 0.214), nor Treatment × Devaluation interaction (*F*_(1,20)_ = 0.28, *P* = 0.603). This implies instrumental memory was not destabilized by the nonreinforced reactivation.

### VR20 reactivation

**Figure 2.**
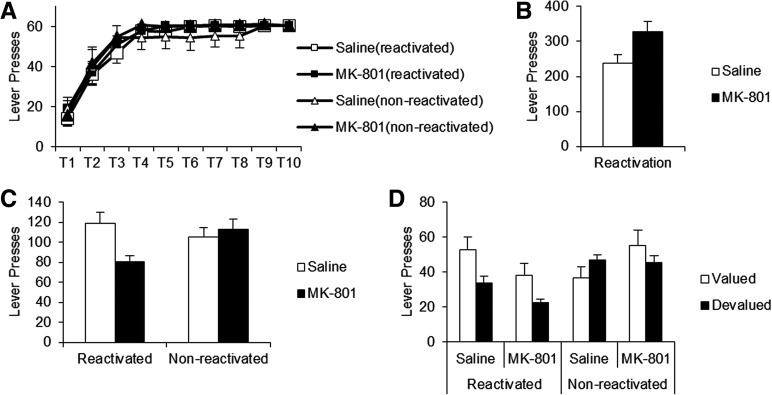
MK-801 significantly impaired lever pressing in a reactivation-dependent manner when given prior to a VR20 reactivation. Switching to a VR20 schedule also rendered behavior sensitive to outcome value, irrespective of drug condition. (*A*) Rats learned to press the lever for food reward over 10 d; performance did not significantly differ between reactivated saline (white squares, *n* = 22) and MK-801 (black squares, *n* = 22), and nonreactivated saline (white triangles, *n* = 10) or MK-801 groups (black triangles, *n* = 10). (*B*) During the VR20 reactivation, MK-801 acutely increased lever pressing performance compared to saline controls. (*C*) When tested 24 h after reactivation, there was a significant reduction of lever pressing performance in VR20-reactivated rats treated with MK-801 (*left*, black bar), compared to saline (white bars) and nonreactivated controls (*right*). (*D*) Following devaluation of the reward pellets (black bars) rats which previously received the VR20 reactivation (*left*, final *n* = 7 per condition) displayed a reduction in lever pressing compared to valued controls (white bars); this effect occurred regardless of prior drug treatment. Nonreactivated rats appeared insensitive to the change in outcome value (*right*, final *n* = 5 per condition). Data expressed as mean ± SEM.

We next tested the efficacy of a VR20 schedule to destabilize instrumental memory, as changes in contingency may be a more reliable method of inducing reconsolidation. All rats increased their lever pressing over Training (*F*_(2.94,176.38)_ = 76.89, *P* < 0.001) (Fig. 2A) with no significant group differences (Treatment, *F*_(1,60)_ = 1.03, *P* = 0.314; Reactivation, *F*_(1,60)_ = 0.02, *P* = 0.903; Treatment × Reactivation, *F*_(1,60)_ = 0.12, *P* = 0.728; Training × Treatment, *F*_(2.94,176.38)_ = 0.19, *P* = 0.900; Training × Reactivation, *F*_(2.94,176.38)_ = 0.81, *P* = 0.488; Training × Treatment × Reactivation, *F*_(2.94,176.38)_ = 0.53, *P* = 0.661).

During the VR20 reactivation, MK-801-injection acutely increased lever pressing (*F*_(1,42)_ = 14.48, *P* < 0.001) (Fig. 2B). When long-term performance was tested 24 h after reactivation (Fig. 2C), ANOVA revealed a significant Treatment × Reactivation interaction (*F*_(1,60)_ = 4.70, *P* = 0.034) with no main effect of Treatment (*F*_(1,60)_ = 2.18, *P* = 0.145) or Reactivation (*F*_(1,60)_ = 0.79, *P* = 0.377).

Analysis of simple main effects revealed a significant reduction in the performance of VR20-reactivated rats injected with MK-801 compared to saline controls (*F*_(1,42)_ = 9.10, *P* = 0.004) (Fig. 2C); however, drug treatment was without effect in the nonreactivated condition (*F*_(1,18)_ = 0.28, *P* = 0.601). Orthogonal analysis of simple main effects showed a significant reduction in lever pressing in MK-801-injected rats given the VR20 reactivation, compared to their nonreactivated counterparts (*F*_(1,30)_ = 8.08, *P* = 0.008). In contrast, there was no significant difference between reactivated and nonreactivated saline controls (*F*_(1,30)_ = 0.57, *P* = 0.455). Thus the overall Treatment × Reactivation interaction at test was driven by the reduction in responding of VR20-reactivated MK-801-treated rats, compared to the reactivated saline group and nonreactivated controls.

Nosepoking behavior was acutely increased by MK-801 during the reactivation session. Although there was no significant difference between treatment groups, nonreactivated controls did make significantly more nosepokes than VR20-reactivated rats during the first extinction test (Supplemental Fig. S4).

In order to test whether the reduction in lever pressing performance represented a loss of instrumental memory we next investigated the sensitivity of behavior to changes in contingency and outcome value (Table 1).

**Table 1.**
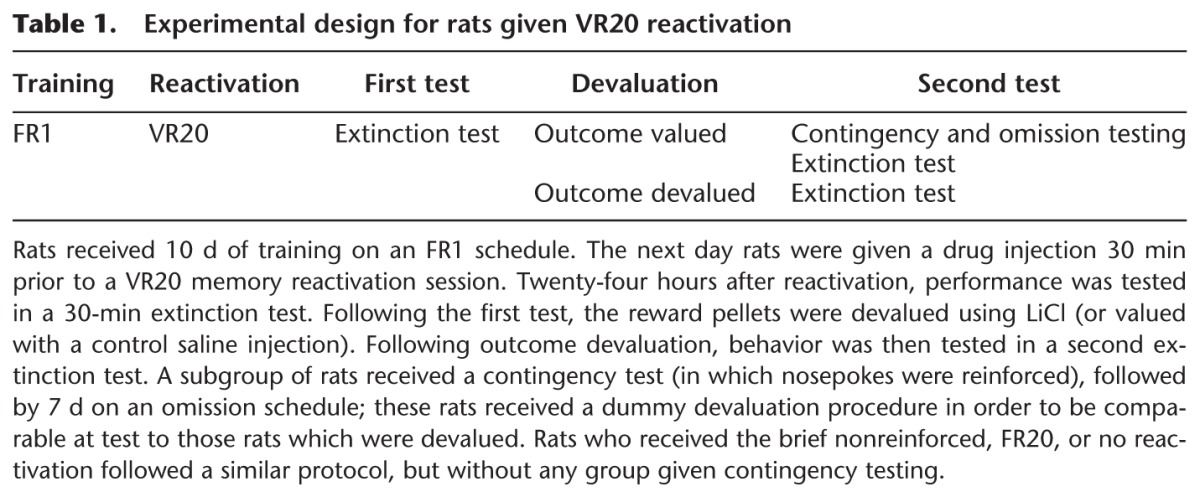
Experimental design for rats given VR20 reactivation

### Reward devaluation

This additional test was intended to determine whether remaining behavior after reconsolidation–disruption was mediated via a representation of the outcome. Each experimental condition was divided into two groups; one group was to have the reward valued, the other devalued by LiCl-pairing. Following outcome devaluation, behavior was retested in extinction (Fig. 2D). ANOVA revealed significant Treatment × Reactivation (*F*_(1,40)_ = 7.03, *P* = 0.011) and Reactivation × Devaluation (*F*_(1,40)_ = 4.64, *P* = 0.037) interactions, with overall main effects of Reactivation (*F*_(1,40)_ = 5.15, *P* = 0.029) and Devaluation (*F*_(1,40)_ = 4.43, *P* = 0.042). These main effects were not a result of preexisting differences between groups (Supplemental Fig. S3B). There was no overall effect of original drug Treatment (*F*_(1,40)_ = 0.35, *P* = 0.558), nor any Treatment × Devaluation (*F*_(1,40)_ = 1.00, *P* = 0.323), nor Treatment × Reactivation × Devaluation interactions (*F*_(1,40)_ = 1.97, *P* = 0.168).

Analysis of simple main effects revealed significantly reduced lever pressing in VR20-reactivated rats previously injected with MK-801, compared to saline controls (*F*_(1,26)_ = 4.47, *P* = 0.044) (Fig. 2D). Nonreactivated controls did not display any significant effect of previous drug treatment on overall performance (*F*_(1,18)_ = 1.95, *P* = 0.179). Orthogonal simple effects revealed a significant reduction in the lever pressing of VR20-reactivated MK-801-injected rats, compared to their nonreactivated MK-801-injected counterparts (*F*_(1,22)_ = 9.76, *P* = 0.005). Rats previously injected with saline vehicle did not show any significant difference in overall lever pressing, regardless of reactivation condition (*F*_(1,22)_ = 0.06, *P* = 0.807). Thus the Treatment × Reactivation interaction was driven by overall lower levels of lever pressing in the VR20-reactivated MK-801-treated group, compared to saline and nonreactivated controls.

Further analysis of simple effects showed that VR20-reactivated rats were sensitive to outcome devaluation (*F*_(1,26)_ = 8.80, *P* = 0.006) (Fig. 2D), but nonreactivated controls did not show any significant reduction in performance following reward devaluation (*F*_(1,18)_ = 0.001, *P* = 0.975). Orthogonal simple effects revealed that lever pressing was lower in VR20-reactivated rats following reward devaluation (*F*_(1,22)_ = 23.23, *P* < 0.001), compared to nonreactivated rats who were similarly subjected to devaluation. Saline-injected rats which still valued the reward pellets did not show any significant difference in performance, regardless of reactivation condition (*F*_(1,22)_ = 0.004, *P* = 0.953). Thus the Reactivation × Devaluation interaction was driven by the sensitivity of the VR20-reactivated rats to the reduction in outcome value.

### Contingency change

The sensitivity of lever pressing to changes in contingency was tested in an additional group of rats. Rats were first reexposed to the reward pellets as in the devaluation procedure, however received only saline injections (thus the reward remained valued). Sensitivity to contingency change was then probed in two tests. Our first contingency test was similar to the VR20 reactivation, however both lever presses and nosepokes were reinforced (with nosepokes reinforced twice as frequently). If behavior was sensitive to outcome contingency, responding should switch from lever pressing to nosepoking.

**Figure 3.**
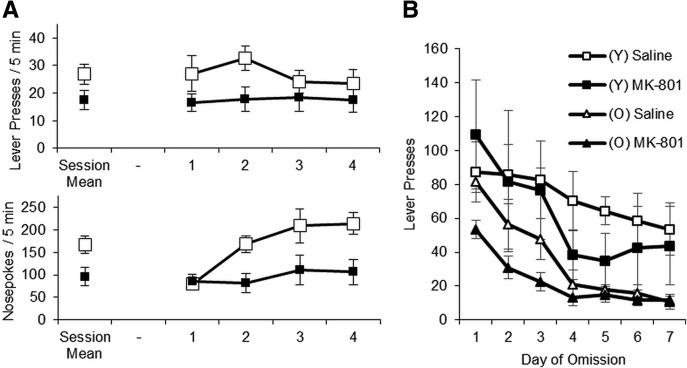
Rats previously injected with MK-801 prior to the VR20 reactivation session displayed a reduced sensitivity to changes in contingency. (*A*) The number of lever presses made by each drug treated group did not significantly differ during the first contingency test (*top*); however, previously saline-injected rats (white squares, *n* = 8) significantly increase their, now rewarded, nosepoking as the session progresses (*bottom*). MK-801-treated rats (black squares, *n* = 8) do not display any significant increase in nosepoke behavior as the session progresses. (*B*) When placed on an omission schedule (O) (triangles), saline-injected rats (white, *n* = 4 per group) significantly reduce their lever pressing compared to yoked controls (Y) (squares) over the 7 d of omission testing. Previously MK-801-treated rats (black, *n* = 4 per group) show no significant difference between omission and yoked groups, and the performance of both diminishes rapidly. Data presented as mean ± SEM.

Analysis of lever presses on this first contingency test revealed no overall difference between saline and MK-801 treated groups (*F*_(1,14)_ = 3.63, *P* = 0.077) (Fig. 3A, top panel), with no significant reduction in performance over the course of the session in either group (Bin, *F*_(1.71,23.89)_ = 0.67, *P* = 0.500; Bin × Treatment, *F*_(1.71,23.89)_ = 0.69, *P* = 0.488).

Analysis of nosepoking during the contingency test revealed a significant Bin × Treatment interaction (*F*_(3,42)_ = 4.54, *P* = 0.020) (Fig. 3A, bottom panel), with significant main effects of Bin (*F*_(3,42)_ = 8.95, *P* < 0.001) and Treatment (*F*_(1,14)_ = 6.31, *P* = 0.025). Analysis of simple main effects revealed a significant increase in nosepoking within the session in saline-injected rats (*F*_(3,21)_ = 11.39, *P* < 0.001), but not MK-801-treated animals (*F*_(1.10,7.70)_ = 0.80, *P* = 0.410). Orthogonal simple effects showed nosepoking in the saline group to be significantly elevated, compared to MK-801-injected rats, in the second and fourth time bins (*F*’s > 8, *P*’s < 0.05), but not in the first and third (*F*’s < 4, *P*’s > 0.05).

In order to investigate further the apparently diminished sensitivity of the VR20-reactivated MK-801 group to contingency change, we divided each treatment group in two. One group was placed on an omission schedule, while the other received yoked pellet deliveries. This enabled comparison of the omission group to yoked controls within each treatment group. Omission and yoked groups were similarly performing prior to omission testing (Supplemental Fig. S5). Over the 7 d of omission, an overall ANOVA revealed a significant Day × Treatment × Omission interaction (*F*_(6,72)_ = 2.46, *P* = 0.032) (Fig. 3B), with significant overall main effects of Day (*F*_(6,72)_ = 20.22, *P* < 0.001) and Omission (*F*_(1,12)_ = 7.03, *P* = 0.021). There was no significant overall main effect of Treatment (*F*_(1,12)_ = 0.73, *P* = 0.409) or any other interactions (Treatment × Omission, *F*_(1,12)_ = 0.01, *P* = 0.931; Day × Treatment, *F*_(6,72)_ = 0.25, *P* = 0.959; Day × Omission, *F*_(6,72)_ = 0.48, *P* = 0.819).

Analysis of simple main effects in saline-treated rats revealed an overall reduction in lever pressing with increasing days on omission (*F*_(6,36)_ = 8.88, *P* < 0.001) (Fig. 3B). Additionally, there was significantly lower responding in saline-treated rats under omission compared to their yoked controls (*F*_(1,6)_ = 8.98, *P* = 0.024), but there was no significant Day × Omission interaction (*F*_(6,36)_ = 1.29, *P* = 0.288). Equivalent analysis of MK-801-injected rats again showed a general reduction in lever pressing as days on omission increased (*F*_(1.81,10.86)_ = 12.03, *P* = 0.002), with no significant difference in performance between rats under omission and yoked controls (Omission, *F*_(1,16)_ = 2.29, *P* = 0.181; Day × Omission, *F*_(1.81,10.86)_ = 1.43, *P* = 0.279).

Orthogonal simple effects showed a significant Day × Treatment interaction in rats under the omission schedule (*F*_(2.27,13.63)_ = 3.95, *P* = 0.040) (Fig. 3B), with a main effect of Day (*F*_(2.27,13.63)_ = 43.22, *P* < 0.001) but not Treatment (*F*_(1,6)_ = 2.65, *P* = 0.154). Yoked controls also showed a significant effect of Day (*F*_(6,36)_ = 5.71, *P* < 0.001). However, there was no difference between treatment groups (Treatment, *F*_(1,6)_ = 1.60, *P* = 0.703; Day × Treatment, *F*_(6,36)_ = 1.13, *P* = 0.365). Further analysis revealed significantly lower lever pressing in MK-801-treated rats under the omission schedule on the first day of omission testing, compared to saline-injected controls also under the omission schedule (*F* > 10, *P* < 0.05). There was no significant effect of Treatment on any other day (all *F*’s < 4, *P*’s > 0.10).

Thus the Day × Treatment × Omission interaction was driven by (1) in saline-treated rats, a significant reduction in lever pressing in the omission group, compared to yoked controls; (2) lower responding in the MK-801 omission group on the first day of omission testing, compared to the saline omission group.

Nosepoke responses did not significantly differ between experimental conditions during the omission testing (Supplemental Fig. S6).

### FR20 reactivation

Following the successful disruption of reconsolidation using the VR20 reactivation, we repeated our experiment using an FR20 reactivation. The FR20 reactivation was intended to investigate whether the salient, reconsolidation-inducing feature of the VR20 reactivation was the change in reward contingency, or the variability inherent within it.

**Figure 4.**
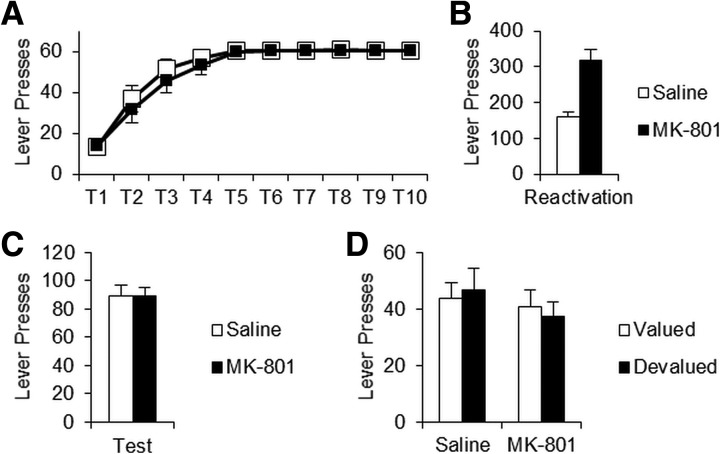
Administration of MK-801 prior to the FR20 reactivation did not impair performance or affect sensitivity to reward devaluation. (*A*) Rats learned to lever press for sucrose reward over the 10 d of training. There were no significant differences between MK-801 (black, *n* = 15) or saline (white, *n* = 14) groups during the training phase. (*B*) MK-801 significantly elevated lever pressing during the FR20 reactivation session compared to saline controls. (*C*) When tested 24 h after reactivation, all rats displayed similar levels of lever pressing regardless of prior treatment. (*D*) Following devaluation of the outcome (black bars: saline, *n* = 7; MK-801, *n* = 8) rats did not display any significant reduction in lever pressing compared to valued controls (white bars: saline, *n* = 7; MK-801, *n* = 7), regardless of drug treatment. Data presented as mean ± SEM.

ANOVA of lever pressing during training revealed a significant increase with Training (*F*_(2.25,60.61)_ = 56.34, *P* < 0.001) (Fig. 4A), with no difference between the treatment groups (Treatment, *F*_(1,27)_ = 0.46, *P* = 0.502; Training × Treatment, *F*_(2.25,60.61)_ = 0.31, *P* = 0.760). During the FR20 reactivation, prior administration of MK-801 significantly augmented lever pressing (*F*_(1,27)_ = 25.36, *P* < 0.001) (Fig. 4B). This effect was only transient; when tested 24 h after reactivation there was no significant effect of Treatment (*F*_(1,27)_ = 0.001, *P* = 0.972) (Fig. 4B).

Nosepoking of MK-801-injected rats was significantly elevated during reactivation, but treatment groups made similar numbers of nosepoke responses during training and testing (Supplemental Fig. S7).

### Reward devaluation

In order to confirm administration of MK-801 prior to the FR20 reactivation had no effect on instrumental memory, we devalued the outcome following the first test, as performed previously. Valued and devalued groups were similarly performing prior to LiCl-pairing (Supplemental Fig. S3C). When retested there was no significant difference between treatment groups (*F*_(1,25)_ = 0.94, *P* = 0.343) (Fig. 4D), nor any overall effect of Devaluation (*F*_(1,25)_ = 0.001, *P* = 0.978), nor Treatment × Devaluation interaction (*F*_(1,25)_ = 0.26, *P* = 0.615).

## Discussion

The present data demonstrate the memories underpinning well-learned lever pressing do undergo reconsolidation, which can be impaired by administration of MK-801. Memory was found to destabilize following a shift in reinforcement contingency to a VR20 schedule; however, administration of MK-801 prior to a brief nonreinforced session, or a shift to FR20, were without effect on behavior. The effect on lever pressing performance with the VR20 reactivation was reactivation-dependent, demonstrating the intervention impaired reconsolidation. Furthermore, the sensitivity of MK-801-treated rats to contingency change appeared diminished following the experimental intervention, suggesting that instrumental memory was impaired. Although the precise associative structure of the remaining behavior is unclear, these data demonstrate that well-learned instrumental memories do destabilize and undergo reconsolidation.

### Reactivation-dependent lever pressing impairment

MK-801 administered prior to the VR20 reactivation successfully disrupted the performance of well-trained lever pressing behavior in a reactivation-dependent manner. Administration of MK-801 in the absence of any behavioral session was without effect, suggesting memory reconsolidation was impaired. Furthermore, the reactivation-dependent reduction in lever pressing was also present on a second extinction test following reward devaluation (6 d after reactivation), providing further evidence that reconsolidation was disrupted, as persistence of amnesia and reactivation-dependence are considered key assessment criteria for reconsolidation impairments (Dudai 2004). MK-801 was without effect when administered prior to a brief nonreinforced or FR20 reactivation, implying these reactivation parameters were insufficient to destabilize lever pressing memory.

One possible alternative interpretation of our data is that the reactivation of the lever pressing memory following MK-801 injection caused subsequent memory retrieval to become state-dependent. Given the absence of MK-801 at test, this would have resulted in the observed impairment, consistent with prior reports that MK-801 causes state-dependent retrieval effects (Ceretta et al. 2008; Flint et al. 2013). However, it is not obvious why the VR20 session, but not the other reactivation procedures, would preferentially induce state-dependent learning. Moreover, the interpretation that post-retrieval memory impairments might reflect state-dependent learning (Millin et al. 2001), importantly, necessitates that a reconsolidation process is taking place such that the destabilized memory trace is restabilized within the current internal state. Thus, while we do not believe it likely that MK-801 impaired lever pressing through state-dependency, rather than disrupting reconsolidation-associated synaptic plasticity, either interpretation of our data strongly concludes that the underlying instrumental memory does undergo reconsolidation.

MK-801 did elevate lever pressing during each reactivation session. However, nosepokes were also increased implying a general motor activation. This is consistent with the acute hyperactivity caused by the doses of MK-801 used here (Hargreaves and Cain 1995). This hyperactivity is only transient, as demonstrated by the lack of any significant long-term augmentation of lever pressing in nonreactivated controls. Moreover, any acute effects of MK-801 cannot explain the reduction in lever pressing following only the VR20 reactivation, as rats given other reactivations showed no long-term deficit. Importantly, the reactivation-dependent reduction in lever pressing with MK-801 was not accompanied by a reduction in nosepoking compared to reactivated saline controls. This would imply that the loss of lever pressing was not simply due to a general motor deficit or any impairment in Pavlovian motivational memories, which are known to undergo reconsolidation (Lee and Everitt 2008; Milton et al. 2012).

### Boundary conditions on induction of reconsolidation

The finding that the reconsolidation of instrumental memory was disrupted contradicts past literature on instrumental reconsolidation, which has suggested instrumental memories do not undergo reconsolidation (Hernandez and Kelley 2004; Mierzejewski et al. 2009). A possible explanation for this discrepancy is that previous literature has used training trials to destabilize the memory, with no change in contingency at reactivation. The present study found a brief nonreinforced or FR20 reactivation did not destabilize memory, and given the likely function of reconsolidation as an updating mechanism (Lee 2009, 2010) it seems unlikely an FR1 reactivation would have produced any effect. Additionally, training trials do not destabilize Pavlovian fear memory when the contingency is well learned (Díaz-Mataix et al. 2013). Importantly, the effect of VR20 was reactivation-dependent and sharply contrasts with previous studies. Thus, the lack of an instrumental effect in past literature appears to be due to the use of inappropriate reactivation parameters.

Notably, MK-801 given prior to the nonreinforced reactivation impaired neither reconsolidation nor extinction. This may be due to the session length, and is consistent with previous studies that have suggested that an intermediate reexposure duration does not behaviorally engage extinction or reconsolidation (Flavell and Lee 2013; Merlo et al. 2014). As such, a still briefer extinction session may prove effective in destabilizing instrumental memory. Interestingly, an FR20 reactivation session also did not destabilize instrumental memory. This would imply that it was not simply exposure to the reward, or reduction in average reward frequency, that caused the instrumental memory to destabilize. Moreover, we might also have expected the exposure to the context and/or reinforcer during the FR20 reactivation to have destabilized Pavlovian memory. The lack of any apparent deficit in Pavlovian memory with any of the reactivation parameters provides further support that the reactivation-dependent effect in the VR20 condition was due to weakening of instrumental memory.

### Prediction errors in reconsolidation

Given that only the VR20 reactivation destabilized instrumental memory, we might infer that only this reactivation produced the putative error signal thought to be necessary to initiate reconsolidation (Lee 2009; Díaz-Mataix et al. 2013; Sevenster et al. 2013). However the brief nonreinforced, and FR20, parameters also represent a change from the training conditions and should also, at least in principle, trigger an error signal. The simplest explanation for this disparity may be that there is a certain threshold for reconsolidation to occur, only exceeded by the VR20. Alternatively, it has been proposed that reconsolidation deficits may be proportional to the prediction-error signal generated (Reichelt and Lee 2013b); the VR20 may have generated a larger error compared to other reactivations.

Interestingly, the FR20 reactivation did not destabilize instrumental memory, despite the fact both the VR20 and FR20 schedules give the same average number of reinforcers, and at least visually produce similar levels of lever pressing in MK-801-treated groups. This raises a question as to why an FR schedule should apparently be insufficient for destabilization to occur, as contingency shifts on a fixed-interval schedule have been used to successfully destabilize Pavlovian fear memory (Díaz-Mataix et al. 2013). A possible answer may lie in the nature of the prediction error signal.

The prediction errors responsible for memory destabilization are believed to originate from dopaminergic neurons (Reichelt et al. 2013), which appear to encode a Temporal Difference (TD) error signal (Sutton and Barto 1998; Glimcher 2011). In this model of prediction error the system predicts future reward deliveries, rather than measuring accumulated reward. Each lever press carries a probability that the next will be reinforced. However, in the VR20 condition lever presses after the first 12 have a diminished predictive value (Supplemental Fig. 8). In the case of Pavlovian memories, a TD error signal appears to be sufficient to destabilize memories (Díaz-Mataix et al. 2013). As such, employing a contingency shift on a fixed-interval schedule may provide a more reliable method of generating TD prediction errors in instrumental settings.

### Sensitivity to outcome value

The VR20 reactivation session not only triggered memory reconsolidation, but also caused subsequent behavior to become sensitive to outcome value, regardless of drug treatment. This effect appears related to the VR20 memory reactivation; no such change in sensitivity to outcome value was observed following the brief nonreinforced or FR20 reactivations. Why such a sensitivity to outcome value should emerge following VR20 is unclear, but may be related to the contingency shift. Contingency of reinforcement is a key determinant in whether behavior is sensitive to outcome value (Dickinson et al. 1983; Wiltgen et al. 2012). Alternatively, resistance of behavior to outcome devaluation appears to be context sensitive (Killcross and Coutureau 2003; Jonkman et al. 2010) and VR20-reactivated rats may have experienced a context change which restored their sensitivity to outcome value. Another explanation may be that destabilization, or reconsolidation, of memory results in, or even requires, the engagement of outcome-encoding processes.

Regardless of its precise cause, the sensitivity of behavior to outcome value alone cannot explain the reactivation-dependent reduction in lever pressing following the VR20 reactivation, as saline controls also display sensitivity to reward devaluation. However, this result is still important, as the ability to display sensitivity to outcome value depends upon intact incentive memory (Balleine and Dickinson 1991; Parkes and Balleine 2013). Incentive memories are known to undergo reconsolidation (Wang et al. 2005) and their loss would theoretically diminish instrumental responding. As both saline and MK-801-injected rats display sensitivity to reward devaluation, and visually there appears to be no difference in the magnitude of this effect, incentive memory is likely intact following the intervention (or at least restored following reexposure to the outcome). Thus, the reduction in lever pressing does not appear to result from any deficit in incentive memory.

### Sensitivity to contingency change

Given that the reactivation-dependent reduction in instrumental behavior likely did not result from any motivational, incentive, or motor deficit, the remaining and most parsimonious explanation for our data is that some aspect of instrumental memory was impaired. Although both saline and MK-801-treated rats had common responses to outcome devaluation, their differential behavior under contingency change and omission training further suggests an MK-801-induced impairment in instrumental memory.

During the contingency change test, both lever presses and nosepokes were reinforced, but with nosepokes reinforced twice as frequently as lever presses. If behavior was sensitive to contingency, one would predict a shift from lever pressing to nosepoking within the session. Although the interpretation is complicated by the preexisting MK-801-induced reduction in lever pressing, the pattern of results showed a clear increase in nosepoke responding through the session in only the saline-treated group. This was accompanied by a small, although statistically nonsignificant, reduction in lever pressing. Importantly, both treatment groups made similar numbers of nosepokes during the prior extinction test, and the first 5 min of the contingency test. Thus it does not appear this effect was simply due to a performance deficit in MK-801-treated rats. Rather, the results appear to indicate a diminished ability of MK-801-treated rats to adapt their instrumental behavior within the session.

Under the omission schedule, all groups reduced their responding over the 7 d. However, only saline-injected rats displayed a significant difference in performance between rats under the omission and yoked controls; MK-801-treated rats showed no significant difference. It is worth noting, however, that the lack of an omission effect in the MK-801 group appears to be driven by a loss of consistent responding in the yoked control group. MK-801-treated rats as a whole began the omission testing at a lower level of responding compared to saline controls. Therefore the lack of a significant omission effect in the MK-801 group may simply represent a floor effect. However, responding in the yoked MK-801 group is generally low after several days of omission testing, and their apparent visually high performance is driven by a single individual. Consequently, the results from the omission schedule support the findings of the previous contingency test, suggesting MK-801-treated rats are less sensitive to changes in instrumental contingency.

### Goal-directed control of responding

By combining the outcome devaluation, contingency change, and omission tests, it is possible to hypothesize about the nature of remaining behavior following the VR20 reactivation in both saline- and MK-801-treated groups. Perhaps the simplest interpretation is within the traditional Belief–Desire framework for assessment of goal-directed behavior (Dickinson and Balleine 1994; de Wit and Dickinson 2009). Experimentally, this hypothesis requires animals to demonstrate sensitivity to changes in contingency (belief) and outcome value (desire). Saline-treated rats appear to fulfil both these criteria following the VR20 reactivation, and thus could be considered goal-directed. On the other hand, while the MK-801-injected group displays sensitivity to outcome value, their sensitivity to contingency appears to be impaired. As a goal-directed R–O association is generally believed to encode the instrumental contingency (Balleine and Dickinson 1998; Yin et al. 2008) it may be that the reconsolidation of this R–O memory was impaired.

Notably, R–O associations are also believed to mediate sensitivity of instrumental behaviors to outcome value. Although we might predict a loss of sensitivity to outcome value if reconsolidation of this memory was impaired, Pavlovian approach also displays sensitivity to outcome value (as demonstrated by the sensitivity of nosepoking to reward devaluation [see Supplemental Fig. S4D; see also Lelos et al. 2011]). It is possible that remaining behavior in MK-801-treated rats is mediated by a Pavlovian conditioned approach to the lever, allowing for lever-contacts of sufficient force to record a lever press. If remaining behavior was Pavlovian this would explain the presence of a devaluation effect in this group. A Pavlovian interpretation is also consistent with the generally accelerated reduction in responding of MK-801-treated rats under both the omission and yoked schedules, as this pattern of data may indicate a greater sensitivity to changes in Pavlovian contingency. Additionally, the diminished ability to acquire the instrumental nosepoke response on the first contingency test may also indicate a greater influence of Pavlovian contingencies; interestingly, there is evidence that strengthening of Pavlovian context associations can impair acquisition of instrumental responding (Dickinson et al. 1996).

It is worth noting that prior to reactivation, behavior was not sensitive to devaluation (see Supplemental Fig. S1), and thus responding was likely habitual following the training phase, consistent with similar training parameters used previously (Adams 1982). As behavior was not sensitive to reward devaluation following the brief nonreinforced, FR20, or no reactivation, it may be that the VR20 reactivation itself functionally disabled habitual responding, leaving behavior under goal-directed control (at least in the saline control group). Consequently, by this interpretation it remains unclear if S–R memories undergo reconsolidation.

One problem with the interpretation presented above is that if behavior in the saline group were goal-directed, and mediated exclusively by an R–O association, we might predict similar baseline levels of performance following reward devaluation to that of the MK-801 group. However, at least visually, this does not appear to be true. Furthermore, this interpretation requires that S–R behavior was disabled in order to explain the performance impairment, otherwise we might have expected S–R responding to compensate for the loss of R–O. This hinges upon R–O and S–R associations being competitive processes (Dickinson et al. 1995), which may not be the case.

An alternative framework for interpreting instrumental behavior proposes that goal-directed and habitual processes are additive (Hogarth and Chase 2011; Hogarth 2012). Briefly, habitual behaviors appear more sensitive to Pavlovian-to-instrumental transfer (PIT) effects (Holland 2004; Wiltgen et al. 2012) and it is well-established that outcome-specific transfer is, paradoxically, insensitive to outcome value (Rescorla 1994; Holland 2004; Corbit et al. 2007). Therefore, actions and habits appear to interact, at least under some circumstances (Balleine and O'Doherty 2010). This hypothesis suggests that R–O associations govern the choice between possible actions, while performance on a certain action is mediated by an S–R association (Hogarth and Chase 2011; Hogarth 2012). This means that the apparent goal-directed nature of responding in saline controls, as assessed through outcome devaluation and contingency changes, is not inconsistent with an intact and functional S–R association. Within such a framework, the present performance deficit in instrumental responding may be interpreted as an impairment in S–R memory reconsolidation.

The appeal of focusing on an S–R impairment is that it may more effectively account for the continued performance deficit at the devaluation test. Were behavior post-VR20 reactivation governed exclusively by an R–O processes it would be expected that devaluation of the outcome should reduce responding in both groups down to a similar baseline level. Moreover, given that both saline- and MK-801-treated rats demonstrated similar sensitivity to outcome devaluation, this could be taken as evidence for intact R–O memory in both groups. Therefore, the performance difference between treatment conditions might be attributable to a deficiency in S–R contribution to instrumental output in MK-801-injected rats.

Specifically, the reconsolidation deficit may be in a habit-like PIT process which contributes to instrumental performance (Hogarth 2012). Outcome-specific PIT is generally believed to occur via a stimulus–outcome–response (S–O–R) associative chain (Balleine and Ostlund 2007; de Wit and Dickinson 2009). Given that nosepoking behavior was not impaired following MK-801-injection, it seems unlikely that the S–O association, mediating the ability of environmental stimuli to predict the outcome, was impaired. It is perhaps more likely that the reconsolidation of a reverse O–R association, which allows the instrumental response to be motivated by the S–O anticipatory process, was disrupted.

It is intuitively obvious how impairment of such a PIT-like process would reduce lever pressing. However, it is unclear why loss of this putative habit association, whatever its associative structure, would lead to a reduced sensitivity to contingency change. This might suggest some aspects of R–O responding were impaired, or at least functionally inhibited, following reconsolidation–disruption. Interestingly, there is some evidence to suggest acquisition of an omission schedule is mediated via an S–R association, which is insensitive to outcome value (Dickinson et al. 1998). As such, S–R associations may make a contribution to contingency learning. Our rather simplistic behavioral design was intended to limit the number of associations being studied from a reconsolidation perspective. This means, however, that it ultimately lacks the sophistication to truly probe the contributions of putative R–O and S–R associations to remaining behavior. Importantly, regardless of the interpretive framework, it remains that the most parsimonious explanation for our data is that the reconsolidation of some aspect of instrumental behavior was impaired (whether that be R–O or S–R), leading to a quantitative reduction in lever pressing.

In summary, we show that the reconsolidation of a well-learned instrumental lever pressing memory can be disrupted by systemic MK-801. Reconsolidation only occurred under specific conditions, and the destabilization of instrumental memory may require a TD prediction error signal. The ability to impair the expression of well-learned instrumental behaviors is potentially of great future clinical benefit in the treatment of maladaptive memory disorders.

## Materials and Methods

### Subjects

Subjects were 117 male Lister-Hooded rats (Charles River), weighing 200–250 g at the start of the experiment. Rats were housed in cages of four at 21°C on a 12-h light–dark cycle (lights on at 07.00) and fed a restricted diet of 15 g/d. Water was available ad libitum. All procedures were approved by a local ethical review committee and carried out in accordance with the United Kingdom 1986 Animals (Scientific Procedures) Act (PPLs 40/3205 & 70/7662).

Twenty-four rats were used in order to test the ability of brief nonreinforced reactivation to destabilize memory. A further 64 rats were used in the VR20 reactivation study and accompanying nonreactivated control, split into MK-801 and saline treated groups. Finally, 29 rats were used for an FR20 reactivation. Groups were initially divided into drug and control treatment groups, which were then each further subdivided into outcome-valued and -devalued groups. A subset of VR20 reactivated rats was given contingency testing, and was subdivided into yoked and omission groups for the omission testing. No rats were excluded from any experimental group.

### Drugs

MK-801 (AbCam) was dissolved in sterile saline to a concentration of 0.1 mg/mL. Thirty minutes prior to memory reactivation, rats were injected intraperitoneally (i.p.) with 0.1 mg/kg of MK-801 or equivalent volume of saline vehicle.

LiCl (Sigma-Aldrich) was dissolved in deionized water to a concentration of 0.12 M. During reward devaluation rats were administered i.p. with 10 mL/kg of LiCl solution, or saline control.

### Instrumental training

Training, reactivation, and testing sessions took place in eight operant boxes (MedAssociates) measuring 25×32×25.5 cm, each housed individually within a soundproof chamber. The rear wall and door were made of Perspex, the other two walls of metal. The boxes contained a grid floor of 19 evenly spaced, stainless steel bars (4.8-mm diameter), underneath which was a removable tray. A nosepoke magazine was mounted on the right-hand wall into which the reward pellets could be delivered, flanked on either side by two retractable levers. The magazine contained an infrared detector which recorded magazine entries. The box was illuminated by a small houselight, mounted on the upper left-hand wall, which came on at the start of each experimental session, switching off at the end. All rats received the same training.

Rats were initially pretrained to collect 45-mg sucrose rodent pellets (5TUL, TestDiet) from the magazine, delivered on a variable-interval (VI) schedule (mean, 60 sec; range, 30–90 sec) for 15 min. The first instrumental training session began immediately after the pretraining session. During training sessions the left lever was extended into the box and presses delivered a reward pellet on an FR1 schedule (one lever press delivered one pellet); the lever did not retract and no reward-paired stimuli were presented during training. Instrumental training sessions lasted 30 min or until a maximum of 60 pellets had been obtained. Rats received a total of 10 training sessions (with a maximum of 60 rewards each) over 10 d.

### Reactivation procedures

Following the 10th day of training, rats were semirandomly divided into two groups, matched for lever pressing performance during training. The day after the end of the training phase rats were administered MK-801, followed 30 min later by a reactivation session.

#### Brief nonreinforced reactivation

A brief extinction session lasted only 5 min. Lever presses were recorded, however no rewards were delivered during this session. Brief nonreinforced sessions have been frequently used to destabilize Pavlovian memories in past studies (Lee et al. 2006; Milton et al. 2008b).

#### VR20 reactivation

This reactivation was similar to training except the reinforcement schedule was shifted to VR20 (mean, 20 presses for one pellet; range, 12–28). The session ended after 20 min, or after a maximum of 20 rewards was obtained. The parameters of this reactivation were based upon our recent finding that weakly trained lever pressing will undergo reconsolidation following a switch to a VR schedule (MT Exton-McGuinness and JL Lee, unpubl.).

#### No reactivation

A control group received drug-injection, but no behavioral session.

#### FR20 reactivation

The experiment was repeated using an FR20 reactivation (20 presses required for a single pellet). The session ended after 20 min, or after a maximum of 20 rewards was obtained.

### Post-reactivation test

Twenty-four hours after reactivation, rats were returned to the operant boxes and their lever pressing performance tested in a 30-min extinction session. The lever was extended, but no pellets were delivered.

### Reward devaluation

In order to test the sensitivity of behavior to changes in outcome value following the different reactivations, the reward pellets were devalued after the initial extinction test by pairing them with LiCl-induced gastric malaise. Devaluation was conducted in two sessions over 2 d. Rats were given free access to the reward pellets for 10 min followed by LiCl or saline injection. After injection, rats were given a further 5 min of access to the pellets before being returned to the home cage. Rats given the brief nonreinforced, FR20, or no reactivation were all then retested the following day in a second 30-min extinction test (6 d after reactivation). Most of the VR20 group was retested as with other groups, however a subgroup received only saline control injections during the devaluation procedure, which was followed by contingency and omission testing instead of an extinction test (see Table 1). For comparison an additional group of rats was trained and received reward devaluation directly following training (Supplemental Fig. S1).

### Contingency change

A set of rats which received the VR20 reactivation were tested for sensitivity to contingency change, following reexposure to the reward pellets (see above). These rats were first given a reinforced session in which the operational contingency was changed. The lever continued to deliver pellets on a VR20 schedule (as in reactivation), however nosepokes into the reward magazine were now also reinforced on a VR10 schedule (mean, 10 nosepokes for one pellet; range, 5–15). This session lasted 20 min.

Although this test produced a large change in nosepoking, effects on lever pressing were minimal. Furthermore, the effect may have been due to preexisting differences between drug groups following reactivation. In order to further investigate, this same group of rats was then placed upon an omission schedule for the next 7 d. During omission testing pellets were delivered on a VI30 schedule (mean, 30 sec; range, 15–45), however lever presses now delayed pellet delivery for a fixed duration of 60 sec. Each rat was paired to a similarly performing partner; one rat received the omission schedule, the other served as a yoked-control. Yoked rats received the same number and frequency of pellet deliveries, however the lever had no programmed consequence. Nosepokes were without consequence during the omission sessions. Omission sessions lasted 30 min.

### Statistical analysis

Training data were analyzed using repeated-measures analysis of variance (ANOVA) in order to assess whether the task was learned and whether experimental groups were equally performing, with Training day and Treatment as factors. The reactivation and test session data were each analyzed separately using one-way ANOVA with drug Treatment as a factor. In the case of the VR20, reactivation data were compared directly with nonreactivated controls with the additional factor of Reactivation in all analyses.

For the devaluation test, data were analyzed with the additional factor of Devaluation. For contingency testing, the initial contingency test was divided into 5-min bins and analyzed with Bin and Treatment as factors. For subsequent testing under omission schedule, data were analyzed with test Day, Omission contingency, and previous drug Treatment as factors. These analyses were also carried out on the prior test in order to assess performance before devaluation (Supplemental Fig. S3) or contingency change (Supplemental Fig. S5).

Equivalent analysis of nosepoking behavior was performed on each session in a similar manner to above, in order to assess general vigor during experimental sessions (see Supplemental Material). In the case of the contingency test, this also served as a measure of acquisition of the new nosepoke contingency.

Results with *P* < 0.05 were deemed significant. A Greenhouse–Geisser correction was used to correct for nonspherical data (as assessed by Mauchley's Test of Sphericity).

## Supplementary Material

Supplemental Material

## References

[EXTON-MCGUINNESSLM035543C1] Adams CD 1982 Variations in the sensitivity of instrumental responding to reinforcer devaluation. Q J Exp Psychol 34B: 77–98

[EXTON-MCGUINNESSLM035543C2] Balleine BW, Dickinson A 1991 Instrumental performance following reinforcer devaluation depends upon incentive learning. Q J Exp Psychol B 43: 279–296

[EXTON-MCGUINNESSLM035543C3] Balleine BW, Dickinson A 1998 Goal-directed instrumental action: contingency and incentive learning and their cortical substrates. Neuropharmacology 37: 407–419 9704982 10.1016/s0028-3908(98)00033-1

[EXTON-MCGUINNESSLM035543C4] Balleine BW, O'Doherty JP 2010 Human and rodent homologies in action control: corticostriatal determinants of goal-directed and habitual action. Neuropsychopharmacology 35: 48–69 19776734 10.1038/npp.2009.131 PMC3055420

[EXTON-MCGUINNESSLM035543C5] Balleine BW, Ostlund SB 2007 Still at the choice-point: action selection and initiation in instrumental conditioning. Ann N Y Acad Sci 1104: 147–171 17360797 10.1196/annals.1390.006

[EXTON-MCGUINNESSLM035543C6] Ceretta APC, Camera K, Mello CF, Rubin MA 2008 Arcaine and MK-801 make recall state-dependent in rats. Psychopharmacology (Berl) 201: 405–411 18758754 10.1007/s00213-008-1304-7

[EXTON-MCGUINNESSLM035543C7] Corbit LH, Janak PH, Balleine BW 2007 General and outcome-specific forms of Pavlovian-instrumental transfer: the effect of shifts in motivational state and inactivation of the ventral tegmental area. Eur J Neurosci 26: 3141–3149 18005062 10.1111/j.1460-9568.2007.05934.x

[EXTON-MCGUINNESSLM035543C8] de Wit S, Dickinson A 2009 Associative theories of goal-directed behaviour: a case for animal–human translational models. Psychol Res 73: 463–476 19350272 10.1007/s00426-009-0230-6 PMC2694930

[EXTON-MCGUINNESSLM035543C9] Díaz-Mataix L, Ruiz Martinez RC, Schafe GE, LeDoux JE, Doyère V 2013 Detection of a temporal error triggers reconsolidation of amygdala-dependent memories. Curr Biol 23: 467–472 23453952 10.1016/j.cub.2013.01.053 PMC3606686

[EXTON-MCGUINNESSLM035543C10] Dickinson A 1985 Actions and habits: the development of behavioural autonomy. Philos Trans R Soc Lond B Biol Sci 308: 67–78

[EXTON-MCGUINNESSLM035543C11] Dickinson A, Balleine BW 1994 Motivational control of goal-directed action. Anim Learn Behav 22: 1–18

[EXTON-MCGUINNESSLM035543C12] Dickinson A, Nicholas DJ, Adams CD 1983 The effect of the instrumental training contingency on susceptibility to reinforcer devaluation. Q J Exp Psychol 35B: 35–51

[EXTON-MCGUINNESSLM035543C13] Dickinson A, Balleine B, Watt A, Gonzalez F, Boakes RA 1995 Motivational control after extended instrumental training. Anim Learn Behav 23: 197–206

[EXTON-MCGUINNESSLM035543C14] Dickinson A, Watt A, Varga ZI 1996 Context conditioning and free operant acquisition under delayed reinforcement. Q J Exp Psychol B 49: 97–110

[EXTON-MCGUINNESSLM035543C15] Dickinson A, Squire S, Varga ZI, Smith JW 1998 Omission learning after instrumental pretraining. Q J Exp Psychol B 51: 271–286

[EXTON-MCGUINNESSLM035543C16] Dudai Y 2004 The neurobiology of consolidations, or, how stable is the engram? Annu Rev Psychol 55: 51–86 14744210 10.1146/annurev.psych.55.090902.142050

[EXTON-MCGUINNESSLM035543C17] Flavell CR, Lee JLC 2013 Reconsolidation and extinction of an appetitive Pavlovian memory. Neurobiol Learn Mem 104: 25–31 23639449 10.1016/j.nlm.2013.04.009

[EXTON-MCGUINNESSLM035543C18] Flint RW, Noble LJ, Ulmen AR 2013 NMDA receptor antagonism with MK-801 impairs consolidation and reconsolidation of passive avoidance conditioning in adolescent rats: evidence for a state dependent reconsolidation effect. Neurobiol Learn Mem 101: 114–119 23391691 10.1016/j.nlm.2013.01.009

[EXTON-MCGUINNESSLM035543C19] Glimcher PW 2011 Understanding dopamine and reinforcement learning: the dopamine reward prediction error hypothesis. Proc Natl Acad Sci 108 (Suppl): 15647–15654 21389268 10.1073/pnas.1014269108 PMC3176615

[EXTON-MCGUINNESSLM035543C20] Hargreaves EL, Cain DP 1995 MK801-induced hyperactivity: duration of effects in rats. Pharmacol Biochem Behav 51: 13–19 7617723 10.1016/0091-3057(94)00321-9

[EXTON-MCGUINNESSLM035543C21] Hernandez PJ, Kelley AE 2004 Long-term memory for instrumental responses does not undergo protein synthesis-dependent reconsolidation upon retrieval. Learn Mem 11: 748–754 15537740 10.1101/lm.84904 PMC534703

[EXTON-MCGUINNESSLM035543C22] Hogarth L 2012 Goal-directed and transfer-cue-elicited drug-seeking are dissociated by pharmacotherapy: evidence for independent additive controllers. J Exp Psychol Anim Behav Process 38: 266–278 22823420 10.1037/a0028914

[EXTON-MCGUINNESSLM035543C23] Hogarth L, Chase HW 2011 Parallel goal-directed and habitual control of human drug-seeking: implications for dependence vulnerability. J Exp Psychol Anim Behav Process 37: 261–276 21500933 10.1037/a0022913

[EXTON-MCGUINNESSLM035543C24] Holland PC 2004 Relations between Pavlovian-instrumental transfer and reinforcer devaluation. J Exp Psychol Anim Behav Process 30: 104–117 15078120 10.1037/0097-7403.30.2.104

[EXTON-MCGUINNESSLM035543C25] Jonkman S, Everitt BJ 2009 Post-learning infusion of anisomycin into the anterior cingulate cortex impairs instrumental acquisition through an effect on reinforcer valuation. Learn Mem 16: 706–713 19864297 10.1101/lm.1497709 PMC2775517

[EXTON-MCGUINNESSLM035543C26] Jonkman S, Everitt BJ 2011 Dorsal and ventral striatal protein synthesis inhibition affect reinforcer valuation but not the consolidation of instrumental learning. Learn Mem 18: 617–624 21921211 10.1101/lm.2269911 PMC3187930

[EXTON-MCGUINNESSLM035543C27] Jonkman S, Kosaki Y, Everitt BJ, Dickinson A 2010 The role of contextual conditioning in the effect of reinforcer devaluation on instrumental performance by rats. Behav Processes 83: 276–281 20060882 10.1016/j.beproc.2009.12.017

[EXTON-MCGUINNESSLM035543C28] Killcross S, Coutureau E 2003 Coordination of actions and habits in the medial prefrontal cortex of rats. Cereb Cortex 13: 400–408 12631569 10.1093/cercor/13.4.400

[EXTON-MCGUINNESSLM035543C29] Lee JLC 2009 Reconsolidation: maintaining memory relevance. Trends Neurosci 32: 413–420 19640595 10.1016/j.tins.2009.05.002 PMC3650827

[EXTON-MCGUINNESSLM035543C30] Lee JLC 2010 Memory reconsolidation mediates the updating of hippocampal memory content. Front Behav Neurosci 4: 168 21120142 10.3389/fnbeh.2010.00168 PMC2991235

[EXTON-MCGUINNESSLM035543C31] Lee JLC, Everitt BJ 2008 Reactivation-dependent amnesia in Pavlovian approach and instrumental transfer. Learn Mem 15: 597–602 18685151 10.1101/lm.1029808 PMC2583130

[EXTON-MCGUINNESSLM035543C32] Lee JLC, Milton AL, Everitt BJ 2006 Reconsolidation and extinction of conditioned fear: inhibition and potentiation. J Neurosci 26: 10051–10056 17005868 10.1523/JNEUROSCI.2466-06.2006 PMC6674482

[EXTON-MCGUINNESSLM035543C33] Lelos MJ, Harrison DJ, Dunnett SB 2011 Impaired sensitivity to Pavlovian stimulus–outcome learning after excitotoxic lesion of the ventrolateral neostriatum. Behav Brain Res 225: 522–528 21871498 10.1016/j.bbr.2011.08.017

[EXTON-MCGUINNESSLM035543C34] McGaugh JL 2000 Memory—a century of consolidation. Science 287: 248–251 10634773 10.1126/science.287.5451.248

[EXTON-MCGUINNESSLM035543C35] Merlo E, Milton AL, Goozee ZY, Theobald DE, Everitt BJ 2014 Reconsolidation and extinction are dissociable and mutually exclusive processes: behavioral and molecular evidence. J Neurosci 34: 2422–2431 24523532 10.1523/JNEUROSCI.4001-13.2014 PMC3921417

[EXTON-MCGUINNESSLM035543C36] Mierzejewski P, Korkosz A, Rogowski A, Korkosz I, Kostowski W, Scinska A 2009 Post-session injections of a protein synthesis inhibitor, cycloheximide do not alter saccharin self-administration. Prog Neuropsychopharmacol Biol Psychiatry 33: 286–289 19100809 10.1016/j.pnpbp.2008.11.015

[EXTON-MCGUINNESSLM035543C37] Millin PM, Moody EW, Riccio DC 2001 Interpretations of retrograde amnesia: old problems redux. Nat Rev Neurosci 2: 68–70 11253361 10.1038/35049075

[EXTON-MCGUINNESSLM035543C38] Milton AL, Everitt BJ 2012 The persistence of maladaptive memory: addiction, drug memories and anti-relapse treatments. Neurosci Biobehav Rev 36: 1119–1139 22285426 10.1016/j.neubiorev.2012.01.002

[EXTON-MCGUINNESSLM035543C39] Milton AL, Lee JLC, Butler VJ, Gardner R, Everitt BJ 2008a Intra-amygdala and systemic antagonism of NMDA receptors prevents the reconsolidation of drug-associated memory and impairs subsequently both novel and previously acquired drug-seeking behaviors. J Neurosci 28: 8230–8237 18701685 10.1523/JNEUROSCI.1723-08.2008 PMC6670560

[EXTON-MCGUINNESSLM035543C40] Milton AL, Lee JLC, Everitt BJ 2008b Reconsolidation of appetitive memories for both natural and drug reinforcement is dependent on β-adrenergic receptors. Learn Mem 15: 88–92 18235109 10.1101/lm.825008

[EXTON-MCGUINNESSLM035543C41] Milton AL, Schramm MJW, Wawrzynski JR, Gore F, Oikonomou-Mpegeti F, Wang NQ, Samuel D, Economidou D, Everitt BJ 2012 Antagonism at NMDA receptors, but not β-adrenergic receptors, disrupts the reconsolidation of Pavlovian conditioned approach and instrumental transfer for ethanol-associated conditioned stimuli. Psychopharmacology (Berl) 219: 751–761 21766171 10.1007/s00213-011-2399-9

[EXTON-MCGUINNESSLM035543C42] Misanin JR, Miller RR, Lewis DJ 1968 Retrograde amnesia produced by electroconvulsive shock after reactivation of a consolidated memory trace. Science 160: 554–555 5689415 10.1126/science.160.3827.554

[EXTON-MCGUINNESSLM035543C43] Nader K 2003 Memory traces unbound. Trends Neurosci 26: 65–72 12536129 10.1016/S0166-2236(02)00042-5

[EXTON-MCGUINNESSLM035543C44] Parkes SL, Balleine BW 2013 Incentive memory: evidence the basolateral amygdala encodes and the insular cortex retrieves outcome values to guide choice between goal-directed actions. J Neurosci 33: 8753–8763 23678118 10.1523/JNEUROSCI.5071-12.2013 PMC3717368

[EXTON-MCGUINNESSLM035543C45] Piñeyro ME, Ferrer Monti RI, Alfei JM, Bueno AM, Urcelay GP 2014 Memory destabilization is critical for the success of the reactivation–extinction procedure. Learn Mem 21: 46–54 24353292 10.1101/lm.032714.113 PMC3867713

[EXTON-MCGUINNESSLM035543C46] Przybyslawski J, Sara SJ 1997 Reconsolidation of memory after its reactivation. Behav Brain Res 84: 241–246 9079788 10.1016/s0166-4328(96)00153-2

[EXTON-MCGUINNESSLM035543C47] Reichelt AC, Lee JLC 2012 Appetitive Pavlovian goal-tracking memories reconsolidate only under specific conditions. Learn Mem 20: 51–60 23263844 10.1101/lm.027482.112

[EXTON-MCGUINNESSLM035543C48] Reichelt AC, Lee JLC 2013a Memory reconsolidation in aversive and appetitive settings. Front Behav Neurosci 7: 118 24058336 10.3389/fnbeh.2013.00118 PMC3766793

[EXTON-MCGUINNESSLM035543C49] Reichelt AC, Lee JLC 2013b Over-expectation generated in a complex appetitive goal-tracking task is capable of inducing memory reconsolidation. Psychopharmacology (Berl) 226: 649–658 23239132 10.1007/s00213-012-2934-3

[EXTON-MCGUINNESSLM035543C50] Reichelt AC, Exton-McGuinness MT, Lee JLC 2013 Ventral tegmental dopamine dysregulation prevents appetitive memory destabilization. J Neurosci 33: 14205–14210 23986254 10.1523/JNEUROSCI.1614-13.2013 PMC6618505

[EXTON-MCGUINNESSLM035543C51] Rescorla RA 1994 Transfer of instrumental control mediated by a devalued outcome. Anim Learn Behav 22: 27–33

[EXTON-MCGUINNESSLM035543C52] Sevenster D, Beckers T, Kindt M 2013 Prediction error governs pharmacologically induced amnesia for learned fear. Science 339: 830–833 23413355 10.1126/science.1231357

[EXTON-MCGUINNESSLM035543C53] Sutton R, Barto A 1998 Reinforcement learning: an introduction. MIT Press, Cambridge, MA

[EXTON-MCGUINNESSLM035543C54] Suzuki A, Josselyn SA, Frankland PW, Masushige S, Silva AJ, Kida S 2004 Memory reconsolidation and extinction have distinct temporal and biochemical signatures. J Neurosci 24: 4787–4795 15152039 10.1523/JNEUROSCI.5491-03.2004 PMC6729467

[EXTON-MCGUINNESSLM035543C55] Wang S-H, Ostlund SB, Nader K, Balleine BW 2005 Consolidation and reconsolidation of incentive learning in the amygdala. J Neurosci 25: 830–835 15673662 10.1523/JNEUROSCI.4716-04.2005 PMC6725620

[EXTON-MCGUINNESSLM035543C56] Wiltgen BJ, Sinclair C, Lane C, Barrows F, Molina M, Chabanon-Hicks C 2012 The effect of ratio and interval training on Pavlovian-instrumental transfer in mice. PLoS One 7: e48227 23144742 10.1371/journal.pone.0048227 PMC3483270

[EXTON-MCGUINNESSLM035543C57] Yin HH, Ostlund SB, Balleine BW 2008 Reward-guided learning beyond dopamine in the nucleus accumbens: the integrative functions of cortico-basal ganglia networks. Eur J Neurosci 28: 1437–1448 18793321 10.1111/j.1460-9568.2008.06422.x PMC2756656

