# Supplementary material for: Reconsolidation of a well-learned instrumental memory
Source: Learn Mem. 2014 Sep;21(9):468–77. doi: 10.1101/lm.035543.114 (PMC4138356; doi:10.1101/lm.035543.114)
Supplement: Supplemental Material [file supp_21_9_468__index.html]

Supplemental Material 

# Reconsolidation of a well-learned instrumental memory

## Supplemental Material

**Files in this Data Supplement:**

- Supplemental Material.pdf
