# Supplementary material for: Reconsolidation of a well-learned instrumental memory
Source: Learn Mem. 2014 Sep;21(9):468–77. doi: 10.1101/lm.035543.114 (PMC4138356; doi:10.1101/lm.035543.114)
Supplement: Supplemental Material [file supp_21.9.468_Supplemental_Material.pdf]

Supplementary Figures

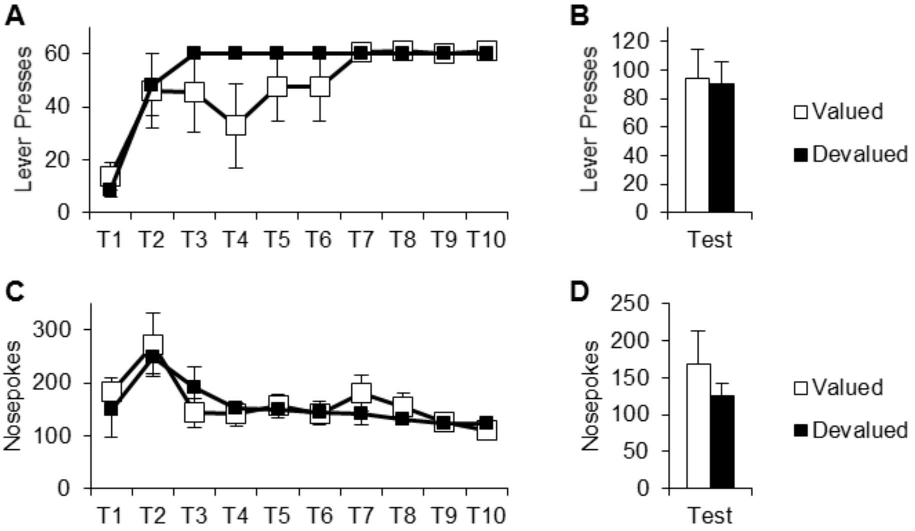

Figure S1: In order to determine whether behaviour was sensitive to outcome value following the training protocol, a cohort of rats received the reward devaluation procedure directly following the training phase. Behaviour was then tested in extinction. Lever pressing was found to be insensitive to changes in outcome value, suggesting training produced habitual responding. **A**, both groups to be valued (white, n=4) and devalued (black, n=4) acquired the lever pressing response to equivalent levels of performance over the 10 days of training (Training: $F_{(2.24,13.46)}$=11.36, p=0.001; Devaluation: $F_{(1,6)}$=0.79, p=0.409; Training x Devaluation: $F_{(2.24,13.46)}$=1.31, p=0.306). A maximum of 60 pellets could be acquired on any one training session. **B**, after training the outcome was devalued and behaviour tested in extinction. Devaluation of the reward pellets did not reduce lever pressing performance compared to valued controls ($F_{(1,6)}$=0.02, p=0.898). **C**, analysis of nosepoking during training revealed a significant reduction in performance over the course of Training ($F_{(2.24,13.45)}$=4.28, p=0.033), but with no group differences (Devaluation: $F_{(1,6)}$=0.21, p=0.661; Training x Devaluation: $F_{(2.24,13.45)}$=0.47, p=0.657). **D**, devaluation of the outcome did not result in a significant reduction in nosepoking during the test session ($F_{(1,6)}$=0.75, p=0.421), although there does appear to be a slight visual reduction compared to valued controls. Data expressed as mean ± SEM.

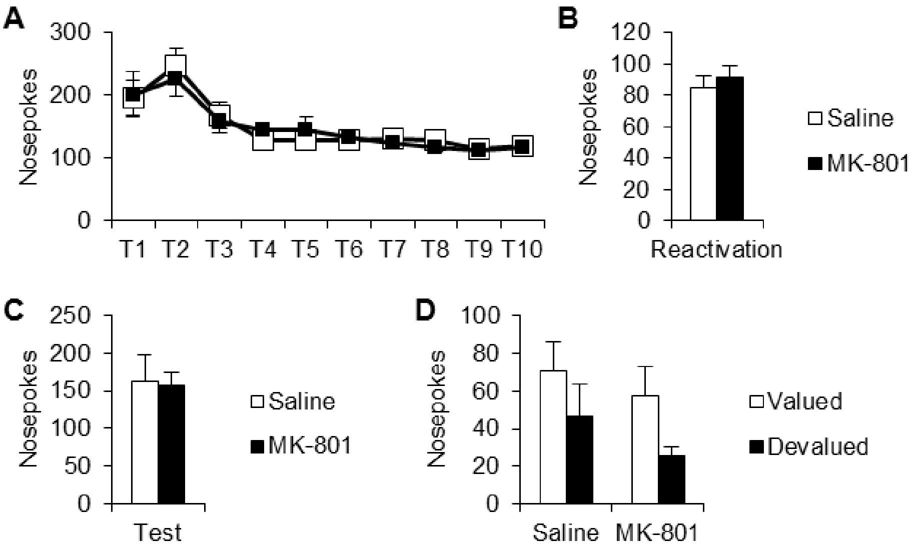

Figure S2: Nosepoke responses did not significantly differ throughout the brief non-reinforced reactivation study. **A**, analysis of nosepoking revealed a significant reduction over the training phase ($F_{(3.36,73.86)}$=11.10, p<0.001) with no difference between saline (white, n=12) or MK-801-treated (black, n=12) experimental groups (Treatment: $F_{(1,22)}$=0.02, p=0.899; Training x Treatment: $F_{(3.36, 73.86)}$=0.25, p=0.882). **B**, nosepoking behaviour did not significantly differ between groups during the brief non-reinforced reactivation ($F_{(1,22)}$=0.45, p=0.510). **C**, both treatment groups displayed similar numbers of nosepoke responses during the first extinction test ($F_{(1,22)}$=0.02, p=0.886). **D**, following the reward devaluation procedure (final n=6 per condition), nosepoking did not significantly differ between experimental conditions (Treatment: $F_{(1,20)}$=1.40, p=0.251; Devaluation: $F_{(1,20)}$=4.00, p=0.059; Treatment x Devaluation: $F_{(1,20)}$=0.07, p=0.788).

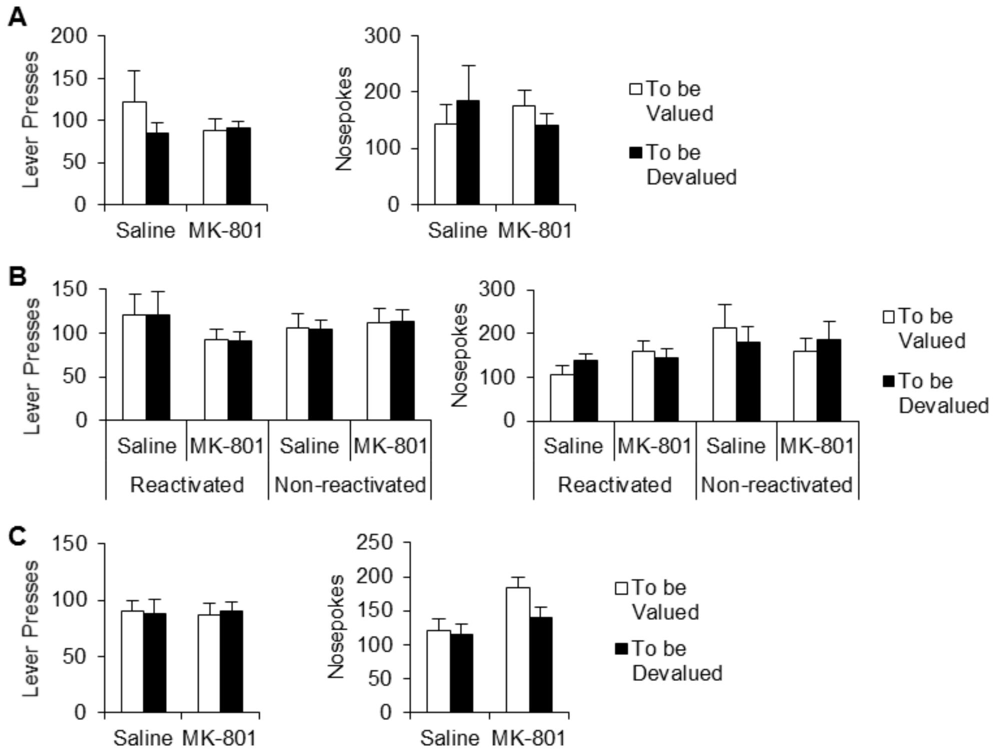

Figure S3: Comparison of valued and devalued groups on the first extinction test, prior to outcome re-exposure. Experimental groups were similarly performing before the devaluation procedure. **A**, following the brief non-reinforced reactivation, there were no significant differences between groups to be valued (white) or devalued (black) in either lever pressing (left; Treatment: $F_{(1,20)}$=0.46, p=0.508; Devaluation: $F_{(1,20)}$=0.68, p=0.420; Treatment x Devaluation: $F_{(1,20)}$=0.89, p=0.357) or nosepoking (right; Treatment: $F_{(1,20)}$=0.02, p=0.889; Devaluation: $F_{(1,20)}$=0.01, p=0.922; Treatment x Devaluation: $F_{(1,20)}$=0.95, p=0.341). **B**, following the VR20 reactivation there were no significant differences in lever pressing between groups to be valued or devalued (left; Treatment: $F_{(1,40)}$=0.68, p=0.415; Reactivation: $F_{(1,40)}$=0.04, p=0.849; Devaluation: $F_{(1,40)}$=0.01, p=0.975; Treatment x Reactivation: $F_{(1,40)}$=1.90, p=0.176; Treatment x Devaluation: $F_{(1,40)}$=0.002, p=0.963; Reactivation x Devaluation: $F_{(1,40)}$=0.001, p=0.982; Treatment x Reactivation x Devaluation: $F_{(1,40)}$=0.007, p=0.933). While non-reactivated groups made significantly more nosepokes than VR20-reactivated rats (see Figure S4C), there were no significant differences in nosepoking between groups to be valued and devalued (right; Treatment: $F_{(1,40)}$=0.02, p=0.898; Reactivation: $F_{(1,40)}$=4.82, p=0.034; Devaluation: $F_{(1,40)}$=0.01, p=0.908; Treatment x

Reactivation: $F_{(1,40)}$=1.43, p=0.239; Treatment x Devaluation: $F_{(1,40)}$=0.01, p=0.910; Reactivation x Devaluation: $F_{(1,40)}$=0.08, p=0.773; Treatment x Reactivation x Devaluation: $F_{(1,40)}$=1.50, p=0.228). **C**, following the FR20 reactivation, there were no significant differences between groups to be valued or devalued in either lever presses (left; Treatment: $F_{(1,25)}$=0.002, p=0.963; Devaluation: $F_{(1,25)}$=0.01, p=0.946; Treatment x Devaluation: $F_{(1,25)}$=0.09, p=0.768) or nosepokes (right; Treatment: $F_{(1,25)}$=2.87, p=0.103; Devaluation: $F_{(1,25)}$=0.69, p=0.415; Treatment x Devaluation: $F_{(1,25)}$=0.06, p=0.816). Data expressed as mean + SEM.

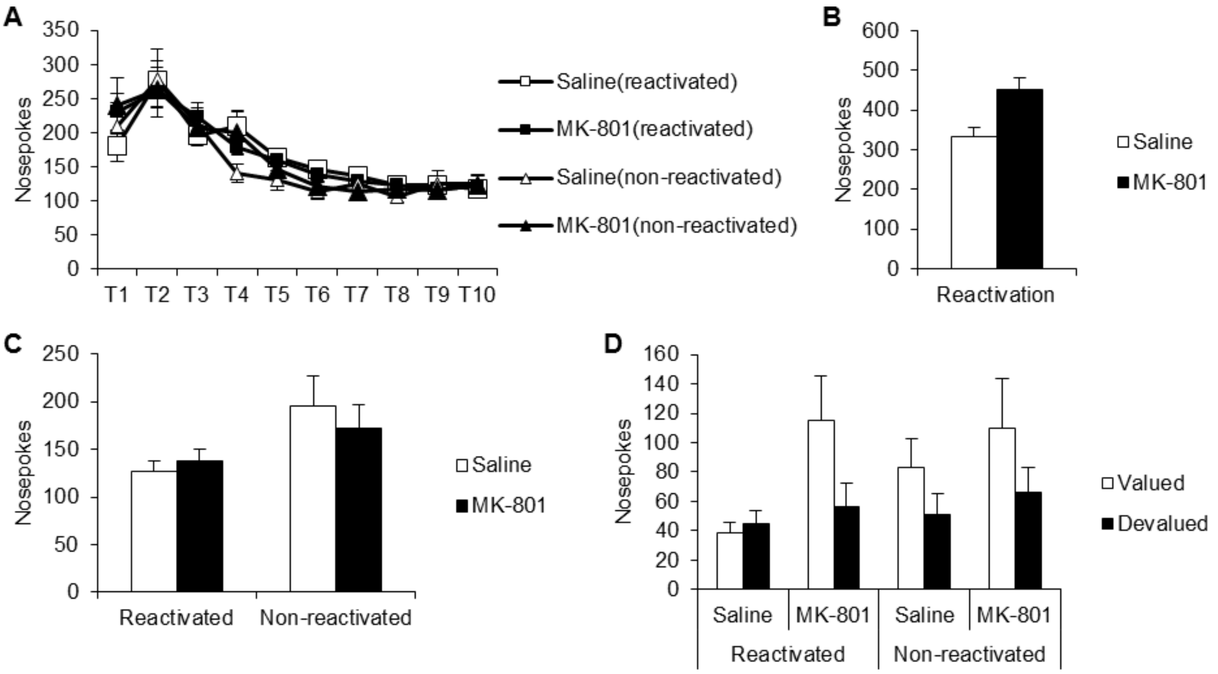

Figure S4: MK-801 caused an acute increase in nosepoking during the VR20 reactivation. The VR20 reactivation also reduced later nosepoke performance at test, regardless of drug condition. **A**, rats reduced their nosepokes over the course of training ($F_{(3.52,211.42)}$=31.49, p<0.001). Rats in reactivated saline (white squares, n=22) and MK-801 groups (black squares, n=22) showed similar levels of nosepoke performance as non-reactivated saline (white triangles, n=10) and MK-801 (black triangles, n=10) controls throughout the training phase (Treatment: $F_{(1,60)}$=0.47, p=0.494; Reactivation: $F_{(1,60)}$=1.12, p=0.295; Treatment x Reactivation: $F_{(1,60)}$=0.34, p=0.563; Training x Treatment: $F_{(3.52,211.42)}$=0.70, p=0.577; Training x Reactivation: $F_{(3.52,211.42)}$=0.63, p=0.622; Training x Treatment x Reactivation: $F_{(3.52,211.42)}$=0.77, p=0.532). **B**, MK-801 injected rats (black bar) displayed an acute increase in nosepoking compared to saline controls (white bar) during the VR20 reactivation ($F_{(1,42)}$=9.15, p=0.004). **C**, when tested 24 hours after reactivation, non-reactivated rats (right) made significantly more nosepokes than reactivated rats (left) at test ($F_{(1,60)}$=8.22, p=0.006), regardless of previous drug treatment (Treatment: $F_{(1,60)}$=0.11, p=0.746; Treatment x Reactivation interaction: $F_{(1,60)}$=0.89, p=0.348). **D**, following devaluation of the sucrose pellets with LiCl, behaviour was re-tested in a second extinction test. On this second test, MK-801-injected rats (reactivated groups, n=7; non-reactivated, n=5) made overall

significantly more nosepokes ($F_{(1,40)}$=5.17, p=0.028) compared to saline-injected controls (reactivated groups, n=7; non-reactivated, n=5); however while visually this effect appears to occur only in reactivated rats, this was not statistically significant (Reactivation: $F_{(1,40)}$=0.97, p=0.330; Treatment x Reactivation: $F_{(1,40)}$=0.68, p=0.416). There was also an overall reduction in nosepoking in rats which received outcome Devaluation ($F_{(1,40)}$=4.97, p=0.031), regardless of drug or reactivation condition (Treatment x Devaluation: $F_{(1,40)}$=1.76, p=0.192; Reactivation x Devaluation: $F_{(1,40)}$=0.17, p=0.684; Treatment x Reactivation x Devaluation: $F_{(1,40)}$=0.85, p=0.362). Data expressed as mean ± SEM.

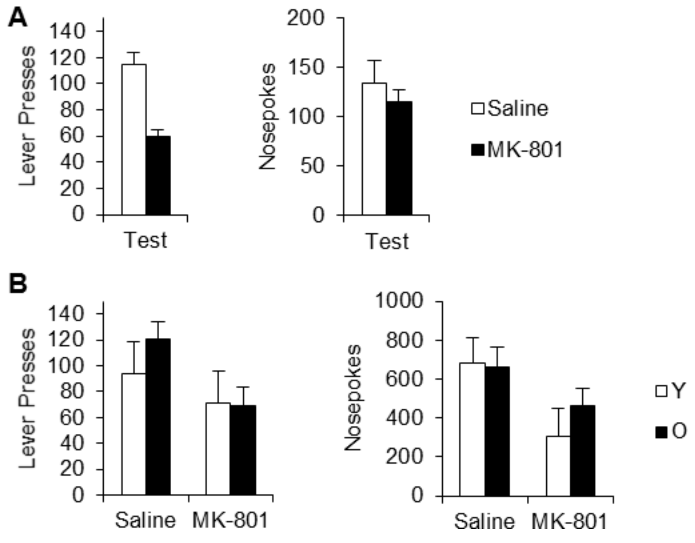

Figure S5: Lever pressing and nosepoke data for the sub-group of VR20 reactivated rats used for contingency and omission testing. **A**, prior to contingency testing, MK-801-treated rats (black bars, n=8) made significantly fewer lever presses compared to saline controls (white bars, n=8) during the first extinction test (left; $F_{(1,14)}$=28.40, p<0.001); however there was no significant difference in nosepoking (right; $F_{(1,14)}$=0.55, p=0.471). **B**, during the contingency test (prior to omission testing) there were no significant differences in lever pressing between the future omission (O; black bars) and yoked (Y; white bars) groups (final n=4 per condition), regardless of previous drug treatment (left; Treatment: $F_{(1,12)}$=3.33, p=0.093; Omission: $F_{(1,12)}$=0.36, p=0.562; Treatment x Omission: $F_{(1,12)}$=0.48, p=0.501). Nosepokes were significantly elevated in the saline-treated group (right; $F_{(1,12)}$=5.78, p=0.033), however yoked and omission groups displayed similar levels of nosepoking within each drug condition (Omission: $F_{(1,12)}$=0.30, p=0.595; Treatment x Omission: $F_{(1,12)}$=0.54, p=0.477). Data expressed as mean + SEM.

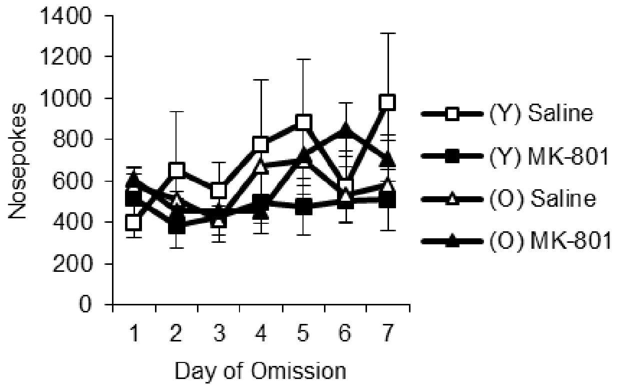

Figure S6: Following the first contingency test, treatment groups were divided in two (final n=4 per condition); one was placed on an omission schedule (O; triangles), while the other acted as a yoked control (Y; squares). Nosepoking during omission testing did not significantly differ on any day between MK-801 (black) and Saline-treated (white) groups, regardless of the reinforcement schedule (Day: $F_{(2.30,27.58)}$=2.92, p=0.064; Treatment: $F_{(1,12)}$=0.46, p=0.509; Omission: $F_{(1,12)}$=0.004, p=0.952; Treatment x Omission: $F_{(1,12)}$=0.99, p=0.339; Day x Treatment: $F_{(2.30,27.58)}$=1.71, p=0.196; Day x Omission: $F_{(2.30,27.58)}$=0.88, p=0.441; Day x Treatment x Omission: $F_{(2.30,27.58)}$=1.18, p=0.329). Data expressed as mean ± SEM.

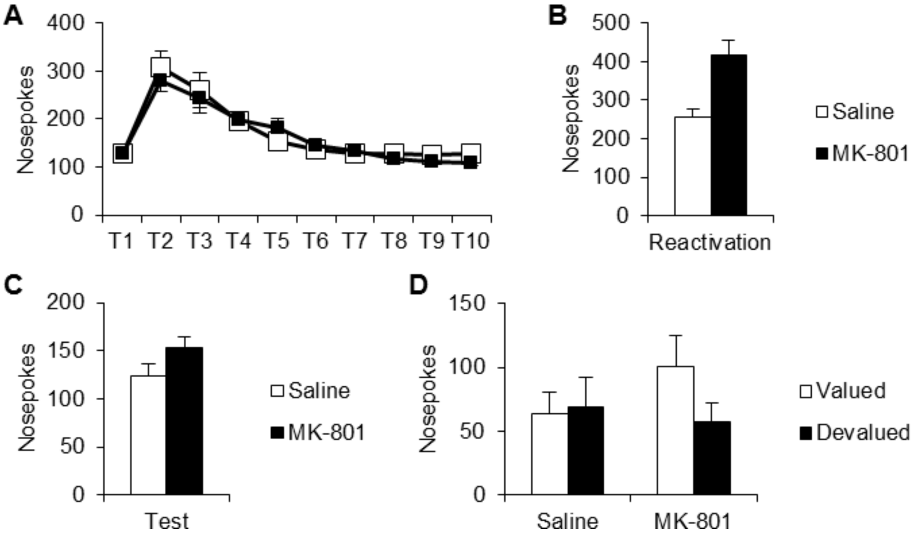

Figure S7: MK-801 acutely increased nosepoking behaviour during the FR20 reactivation session, however did not result in any significant long-term effect on behaviour. **A**, nosepokes significantly reduced over the course of training ($F_{(2.77,74.70)}$=23.52, p<0.001), however there were no differences between saline (white squares, n=14) or MK-801-treated (black squares, n=15) groups (Treatment: $F_{(1,27)}$=0.45, p=0.510; Training x Treatment: $F_{(2.77,74.70)}$=0.43, p=0.720). **B**, during the FR20 reactivation session, rats injected with MK-801 (black bars) significantly increased their nosepoking behaviour ($F_{(1,27)}$=15.26, p=0.001) compared to saline controls (white bars). **C**, when tested in extinction the day following reactivation, there was no significant difference in the number of nosepokes between treatment groups ($F_{(1,27)}$=2.89, p=0.101). **D**, nosepokes were not significantly affected by devaluation of the reward pellets (black bars), compared to valued controls (white bars). While MK-801-treated rats appear to show a moderate visual devaluation effect, this was not significant (Treatment: $F_{(1,25)}$=0.54, p=0.469; Devaluation: $F_{(1,25)}$=0.92, p=0.348; Treatment x Devaluation: $F_{(1,25)}$=0.16, p=0.689). Data expressed as mean ± SEM.

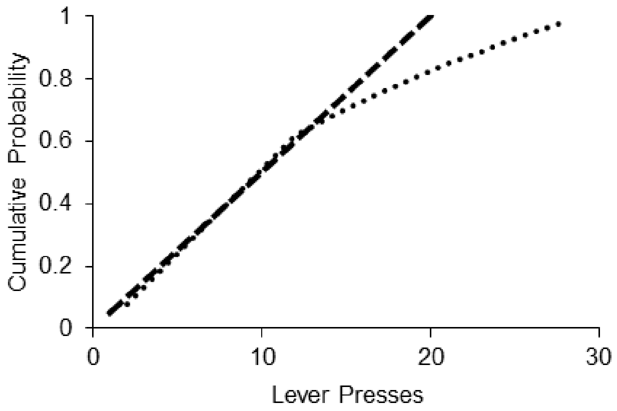

Figure S8: Cumulative probability of reinforcement on each subsequent lever press during the FR20 (dashed line) and VR20 reactivation (dotted line). The first 12 lever presses on each schedule offer similar predictions over whether future lever presses are reinforced. Each subsequent lever press under the VR20 schedule carries a lower predictive value over future reinforcer delivery.
